# Dyssegmental dysplasia Rolland–Desbuquois type is caused by pathogenic variants in *HSPG2* - a founder haplotype shared in five patients

**DOI:** 10.1038/s10038-024-01229-6

**Published:** 2024-02-29

**Authors:** Paniz Farshadyeganeh, Takahiro Yamada, Hirofumi Ohashi, Gen Nishimura, Hiroki Fujita, Yuriko Oishi, Misa Nunode, Shuku Ishikawa, Jun Murotsuki, Yuri Yamashita, Shiro Ikegawa, Tomoo Ogi, Eri Arikawa-Hirasawa, Kinji Ohno

**Affiliations:** 1 Division of Neurogenetics, Center for Neurological Diseases and Cancer, Nagoya University Graduate School of Medicine, Nagoya, Japan; 2 Division of Clinical Genetics, Hokkaido University Hospital, Sapporo, Japan; 3 Division of Medical Genetics, Saitama Children’s Medical Center, Saitama, Japan; 4 Department of Radiology, Musashino Yowakai Hospital, Tokyo, Japan; 5 Department of Orthopaedics, Hokkaido Medical Center for Child Health and Rehabilitation, Sapporo, Japan; 6 Department of Obstetrics, Asahikawa Medical University, Asahikawa, Japan; 7 Department of Obstetrics, Osaka Medical and Pharmaceutical University, Takatsuki, Japan; 8 Department of Neonatal Internal Medicine, Hokkaido Medical Center for Child Health and Rehabilitation, Sapporo, Japan; 9 Department of Maternal and Fetal Medicine, Miyagi Children’s Hospital, Sendai, Japan; 10 Aging Biology in Health and Disease, Juntendo University Graduate School of Medicine, Tokyo, Japan; 11 Research Institute for Diseases of Old Age, Juntendo University Graduate School of Medicine, Tokyo, Japan; 12 Center for Integrative Medical Sciences, RIKEN, Tokyo, Japan; 13 Department of Genetics, Research Institute of Environmental Medicine (RIeM), Nagoya University, Nagoya, Japan

**Keywords:** Genetics research, Disease genetics

## Abstract

Dyssegmental dysplasia (DD) is a severe skeletal dysplasia comprised of two subtypes: lethal Silverman–Handmaker type (DDSH) and nonlethal Rolland–Desbuquois type (DDRD). DDSH is caused by biallelic pathogenic variants in *HSPG2* encoding perlecan, whereas the genetic cause of DDRD remains undetermined. Schwartz–Jampel syndrome (SJS) is also caused by biallelic pathogenic variants in *HSPG2* and is an allelic disorder of DDSH. In SJS and DDSH, 44 and 8 pathogenic variants have been reported in *HSPG2*, respectively. Here, we report that five patients with DDRD carried four pathogenic variants in *HSPG2*: c.9970 G > A (p.G3324R), c.559 C > T (p.R187X), c7006 + 1 G > A, and c.11562 + 2 T > G. Two patients were homozygous for p.G3324R, and three patients were heterozygous for p.G3324R. Haplotype analysis revealed a founder haplotype spanning 85,973 bp shared in the five patients. SJS, DDRD, and DDSH are allelic disorders with pathogenic variants in *HSPG2*.

## Introduction

Dyssegmental dysplasia (DD) is a severe skeletal dysplasia characterized by dumbbell-shaped tubular bones, bent long bones of the legs, and irregular size and shape in single or multiple vertebral ossification centers (anisospondyly). DD is comprised of two subtypes [1]: lethal Silverman–Handmaker type (DDSH) [2] and nonlethal Rolland–Desbuquois type (DDRD) [3]. DDSH and DDRD are spectrum disorders, and the differences of skeletal phenotypes are not crystal clear. In general, anisospondyly is much more severe in DDSH than in DDRD. Some vertebral bodies are rudimentary or even absent in DDSH, while all vertebral bodies are better ossified in DDRD. The mildest end of DDRD is associated with only large coronal clefts at the thoracolumbar spine, as is Kniest dysplasia (a rare variant of *COL2A1*-linked skeletal dysplasias) [4]. DDSH is sometimes accompanied by hydrocephalus and occipital encephalocele that may manifest with defective calvarial ossification, while DDRD is not. However, calvarial ossification defects are not the norm for DDSH. DDSH is inherited in an autosomal recessive manner and is caused by pathogenic variants in *HSPG2* encoding perlecan [5, 6], whereas pathogenic variants have not been reported in DDRD. Schwartz–Jampel syndrome (SJS) is also caused by pathogenic variants in *HSPG2* and is inherited in an autosomal recessive manner. Thus, SJS is an allelic disorder of DDSH. In SJS and DDSH, 44 and 8 pathogenic variants have been reported in *HSPG2*, respectively (Table 1). Partial lack of perlecan is likely to cause SJS, whereas complete or almost complete lack of perlecan is likely to cause DDSH [7].

**Table 1:** Previously reported pathogenic variants in *HSPG2* in SJS and DDSH

Coordinate (GRCh38)	NM_005529.7	NP_005520.4	Region/Domain^a^	dbSNP	HGMD	InMeRF score^b,13^	CADD score^b,32^	DANN score^b,33^	Ref.
Schwartz–Jampel syndrome (SJS)									
21,889,500	c.574 + 481 C > T	splicing		rs916959204	CS065568	na	na	na	[7]
21,887,966–21,887,976	c.665_675del	frameshift		na	CD065741	na	na	na	[7]
21,884,528–21,887,623	c.720_1654del	frameshift		na	na	na	na	na	[7]
21,885,405	c.1125 C > G	p.C375W	4th LDL receptor type A/Domain II	rs543805444	na	0.965	0.382	0.377	[36]
21,884,877	c.1356–10 G > A	splicing		na	na	na	na	na	[37]
21,884,513	c.1654 + 15 G > A	splicing		rs886046043	CS1312769	na	na	na	[38]
21,876,592	c.2746 C > T	p.R916X		na	CM1312167	na	na	na	[38]
21,876,497	c.2826 + 15 G > A	splicing		na	na	na	na	na	[38]
21,875,990	c.3056 C > T	p.P1019L	2nd laminin type B/Domain III	rs62642528	CM065274	0.595	0.319	0.599	[7]
21,875,668	c.3263 T > C	p.L1088P	↓	na	CM157180	0.335	0.626	0.931	[39]
21,872,996	c.3888 + 1 G > A	splicing		na	na	na	na	na	[40]
21,865,037	c.4432 C > T	p.R1478C	3rd laminin type B/Domain III	rs1198712778	CM065272	0.515	0.593	0.992	[7]
21,864,994–21,864,996	c.4473_4475del	inframe		na	CD065740	na			[7]
21,864,874	c.4595 G > A	p.C1532Y	Interface between 3rd laminin type B and 7th laminin EGF-like/Domain III	rs137853248	CM003145	0.966	0.647	0.749	[5]
21,864,192	c.4648 C > T	p.R1550C	↓	rs1317669197	CM065277	0.741	0.705	0.997	[7]
21,864,100	c.4740 G > A	splicing		na	CS003180	na	na	na	[5]
21,864,095	c.4740 + 5 G > A	splicing		rs886039909	CS1613304	na	na	na	[41]
21,862,125	c.4741–10 T > G	splicing		na	CS065566	na	na	na	[7]
21,855,680	c.5702–5 G > A	splicing		rs2290498	CS1514375	na	na	na	[42]
21,854,720	c.6179delC	frameshift		na	CD065739	na	na	na	[7]
21,851,790	c.7006 + 1 G > A	splicing		rs778653296	CS065567	na	na	na	[7]
21,850,359	c.7294 + 4 A > G	splicing		na	CS021002	na	na	na	[43]
21,847,842	c.7874–2 A > G	splicing		rs931293134	CS065569	na	na	na	[7]
21,847,960–21,847,840	Fusion of exons 60 and 61	splicing		na	na	na	na	na	[43]
21,846,108	c.8464 G > A	Splicing (last exonic nucleotide)		rs748523693	CS021003	na	na	na	[40]
21,846,104	c.8464 + 4 A > G	splicing		rs1572204991	CS003181	na	na	na	[5]
21,843,375	c.8680 C > T	p.Q2894X		rs1004543320	CM065273	na	na	na	[7]
21,842,916–21,842,924	c.8759–3_8764del	inframe		na	CD021017	na	na	na	[43]
21,842,892	c.8788 G > A	p.E2930K	Interface between 13th and 14th immunoglobulin-like regions/Domain IV	rs368020528	CM187975	0.286	0.618	0.962	[44]
21,842,014	c.9181 C > T	p.Q3061X		na	CM157183	na	na	na	[39]
21,841,541	c.9326delA	frameshift		rs2098048505	CD065736	na	na	na	[7]
21,839,888	c.9643delC	frameshift		na	CD065735	na	na	na	[7]
21,836,803	c.10354 C > T	p.R3452X		rs1208167285	CM065275	na	na	na	[7]
21,836,802	c.10355 G > A	p.R3452Q	19th immunoglobulin-like region/Domain IV	rs1327754652	CM065276	0.428	0.711	0.989	[7]
21,833,870	c.10776delT	frameshift		na	CD1514376	na	na	na	[42]
21,833,381	c.10982 G > A	p.R3661Q	Interface between 21st immunoglobulin-like region and 1st laminin type G/Domains IV and V	na	na	0.489	0.520	0.988	
21,833,363	c.11000 C > T	p.T3667M	1st laminin type G/Domain V	rs369084217	CM1821688	0.571	0.943	0.968	[14]
21,832,510	c.11192delG	frameshift		na	CD065738	na	na	na	[7]
21,832,495	c.11207 G > A	p.R3736Q	1st laminin type G/Domain V	rs984839674	CM1911787	0.690	0.800	0.997	[45]
21,831,803	c.11208–7 G > A	splicing		na	na	na	na	na	[7]
21,829,582–21,829,583	c.11792dupC	frameshift		na	CI065834	na	na	na	[7]
21,828,878	c.12194delC	frameshift		na	CD065737	na	na	na	[7]
21,824,120	c.12899 + 1 G > A	splicing		na	na	na	na	na	[7]
21,816,592–21,823,699	c.12920del7108	splicing		na	na	na	na	na	[43]
Dyssegmental dysplasia, Silverman–Handmaker type (DDSH)									
21,887,995	c.646 G > T	p.E216X		na	CM1310600	na	na	na	[29]
21,873,006–21,873,009	c.3876_3879del	frameshift		na	CD106077	na	na	na	[28]
21,872,619	c.4029 + 1 G > A	splicing		rs779249304	CS188869	na	na	na	[30]
21,855,589	c.5788 C > T	p.Q1930X		na	CM1310601	na	na	na	[29]
21,851,786	c.7006 + 5 G > A	splicing		rs1208832174	CS011045	na	na	na	[6]
21,839,005	c.9970 G > A	p.G3324R	18th immunoglobulin-like region/Domain IV	rs1294413650	CM1821690	0.752	0.927	0.989	[14]
21,833,551	c.10894 C > T	p.R3632X		rs762281715	CM1821689	na	na	na	[14]
21,824,603–21,824,604	c.12666‑2_12677dup	frameshift		na	CI2015701	na	na	na	[31]

*splicing* aberrant splicing is induced, *na* not available or not applicable

^a^Domains are according to RefSeq NM_001291860.2, and are indicated only for missense variants. Please also refer to Fig. 2A for schematic presentation

^b^The most pathogenic score is 1.000 and the least pathogenic score is 0.000 [13, 32, 33]

In five patients with DDRD, we identified pathogenic variants in *HSPG2*. Haplotype analysis revealed that p.G3324R in *HSPG2* had a founder haplotype shared in all the five patients.

## Patients and methods

### Patients

All the human studies were approved by the institutional review board of Nagoya University Graduate School of Medicine (Approval #2007-0598). Appropriate written informed consents were obtained from all the participated patients/guardians and parents.

### Whole exome resequencing analysis

Genomic DNA was isolated from the blood with QIAamp Blood Mini Kit (Qiagen) according to the manufacturer’s instruction. Next-generation sequencing (NGS) was performed on the Illumina HiSeq platform. In a patient labeled as DDRD_P02, pathogenic variants were searched for by whole-genome sequencing. The Illumina adapter-ligated gDNA fragments were sequenced using paired-end (PE) flow cells and 2 × 151 bp raw fastq data were obtained. In the other four patients, pathogenic variants were looked for by whole exome sequencing. gDNA was enriched by using the Agilent SureSelect Human All Exon Kit version 6. The captured exonic fragments were sequenced on PE flow cells and 2 × 151 bp raw fastq data were obtained. The fastq data were analyzed by our standard NGS pipelines [8]. Briefly, low-quality reads and sequencing adapters were removed by fastp version 0.23.4 [9]. The reads were then aligned to the human reference genome (GRCh37) using the Burrows–Wheeler Aligner (bwa) version 0.7.12-r1039 (https://arxiv.org/abs/1303.3997). PCR duplicates were removed by bammarkduplicate2 version 2.0.72 [10]. The mapped reads were then locally realigned and base quality scores were recalibrated using Genome Analysis Toolkit (GATK 3.5, IndelRealigner, and BaseRecalibrator) [11]. Single-nucleotide variants (SNVs) were identified by the GATK HaplotypeCaller. The identified variants were annotated by ANNOVAR [12]. All the identified pathogenic variants were confirmed by Sanger sequencing in patients and their available parents using PCR primers (Supplementary Table S1). PCR products were purified using the Wizard SV Gel and PCR Clean-Up System (Promega) according to the manufacturer’s protocols. Sanger sequencing was performed on 3730xl DNA Analyzer (Thermo Fisher Scientific).

### Haplotype analysis

SNVs and small indels spanning 5,000,000 bp on both sides of p.G3324R were collated from the whole-genome-seq of DDRD_P02 and the exome-seq of DDRD_P03, P04, P06, P07, and P08. An uninterrupted segment of homozygous SNVs/indels were first searched for from p.G3324R in DDRD_P02 and then in DDRD_P03. The SNVs/indels in the putative shared haplotypes were then searched for in DDRD_P06, P07, and P08. DDRD_P06, P07, and P08 were heterozygous for p.G3324R, and exome-seq data were not available in their parents. Thus, we could not determine whether an SNV/indel was on the same allele as p.G3324R or not. When an SNV/indel was present in DDRD_P06, P07, and P08 in a heterozygous manner, we assumed that the SNV/indel was on the same allele as p.G3324R. The putative shared haplotype was thus narrowed down using exome-seq data of DDRD_P06, P07, and P08.

## Results

### Clinical features of five patients

**Fig. 1:**
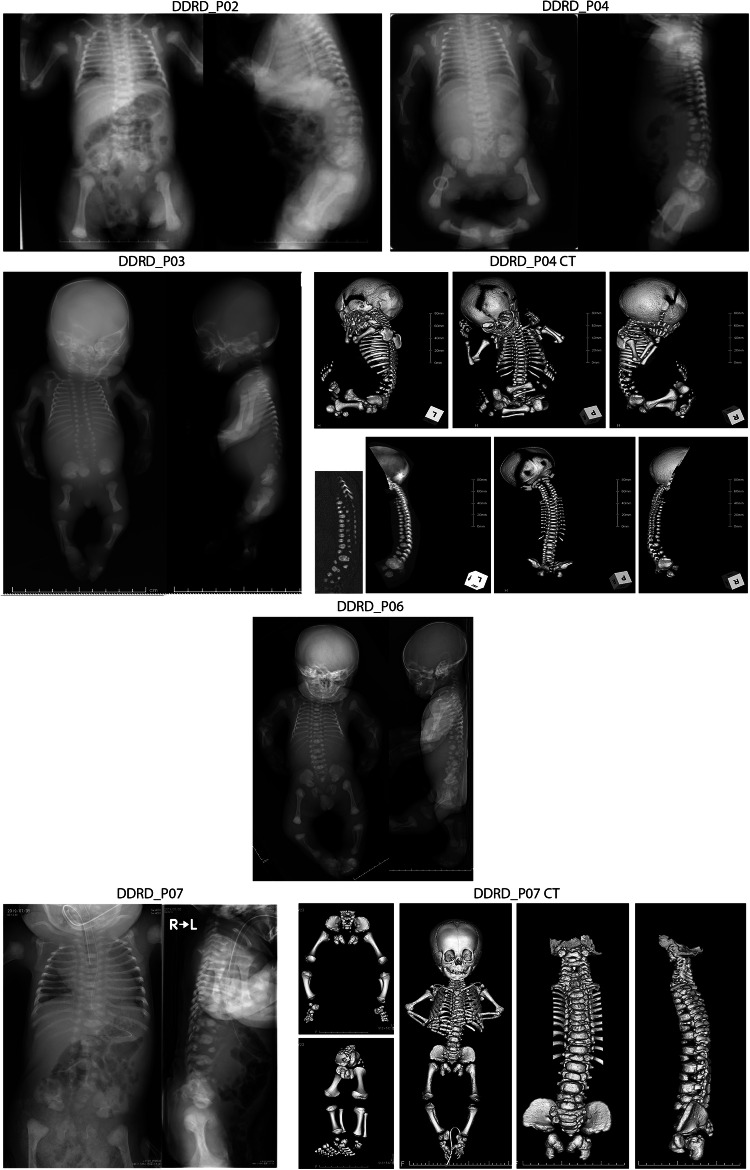
Representative X-ray images and reconstructed CT images of DDRD_P02, P03, P04, P06, and P07

DDRD_P02 (male) was noted of a cross-legged position at 19 weeks of gestation, but the femur length was not shortened then (−0.84 SD). Thereafter, the femur lengths were −1.6 SD at 22 weeks, −1.9 SD at 26 weeks, −2.3 SD at 28 weeks, and −2.9 SD at 31 weeks. As the femur lengths exceeded −2.5 SD at 31 weeks, skeletal dysplasia was extensively evaluated. At 31 weeks, irregular ossification of the vertebral bodies was also identified (Fig. 1). Both 3D-US and 3D-CT delineated the diagnostic features of DD, including dumbbell deformity of the tubular bones, bowing of the long bones, and anisospondyly. However, dumbbell deformity was not conspicuous, and bowing was very mild. The patient showed extensive anisospondyly, but the constellation of the overall imaging findings was consistent with DDRD not DDSH. The birth weight was 2744 g at 37 weeks. He had respiratory failure that required respiratory support for 49 days after birth. The parents were not consanguineous, and no similar patients were reported in the family.

DDRD_P03 (male) presented with proximal dominant shortening of long bones, fracture-like deformities of the femurs, bilateral clubfoot, narrowing of the thorax, and abnormal curvature of the spine at 19 weeks of gestation (Fig. 1). Defective thoracic vertebral segmentation was also noted by fetal US examination then. The biparietal diameter was +3.7 SD, the femur length was −4.0 SD, and the humerus length was −1.6 SD. Pregnancy was terminated at 20 weeks and 6 days of gestation due to the diagnosis of severe skeletal dysplasia. The body weight was 440 g. The termination of pregnancy made it difficult to discern by X-ray whether the patient fitted better to either DDSH or DDRD. The parents were not consanguineous, and no similar patients were reported in the family.

DDRD_P04 (female) presented with moderate shortening of the long bones: femur length of −4.2 SD and humerus length of −4.3 SD at 30 weeks of gestation (Fig. 1). Both 3D-US and 3D-CT supported the diagnosis of DD with findings of dumbbell deformity of the tubular bones, bowing of the long bones, and anisospondyly. The mild to moderate degrees of skeletal dysplasia supported the diagnosis of DDRD rather than DDSH. The patient was born at 35 weeks with birth weight of 2374 g. She had respiratory insufficiency that required respiratory support for 44 days after birth. She also had hypoplastic thorax, cleft palate, restricted limb joint movements. She had mild bilateral blepharophimosis, mild pursed lips, and mild limb myotonia, which are commonly observed in SJS. She is currently 11 years and 11 months old. The parents were not consanguineous, and no similar patients were reported in the family.

DDRD_P06 (male) was diagnosed of short long bones in all four limbs (−4 to −5 SD) at 28 weeks of gestation. The lower limbs were in a cross-legged position. CT scan showed marked anisospondyly and mild dumbbell-shaped tubular bones (Fig. 1). In contrast to severe spinal deformities, shortening and bowing of long bones were mild, which supported the diagnosis of DDRD. At 38 weeks of gestation, the patient was delivered in breech position with the body weight of 2299 g. The patient died several hours after birth due to respiratory failure. Respiratory support was not applied. The parents were not consanguineous, and no similar patients were reported in the family.

DDRD_P07 (male) was noted to have short limbs and inguinal herniation in fetal US examination. Prenatal diagnosis of DD was not made. The birth weight at 38 weeks of gestation was 2626 g (−0.6 SD) with a height of 45 cm (−1.78 SD). The Apgar scores were 3 and 7 points at 5 and 10 min, respectively. He required respiratory support after birth. At 6 months of age, he became independent on a respirator during the day. At 10 months of age, he had flat nasal root, marked micrognathia, U-shaped cleft palate, low-set ears, short neck, small thorax, short limbs especially in humeri and femurs, curved lower legs, clubfeet, inguinal herniation, and cryptorchidism. Anisospondyly consistent with DDRD was noted on X-ray (Fig. 1). He was deceased at age 3 years and 8 months because of accidental extubation of the tracheostomy cannula at night at home. The parents were not consanguineous, and no similar patients were reported in the family.

### Identification of pathogenic variants

**Fig. 2:**
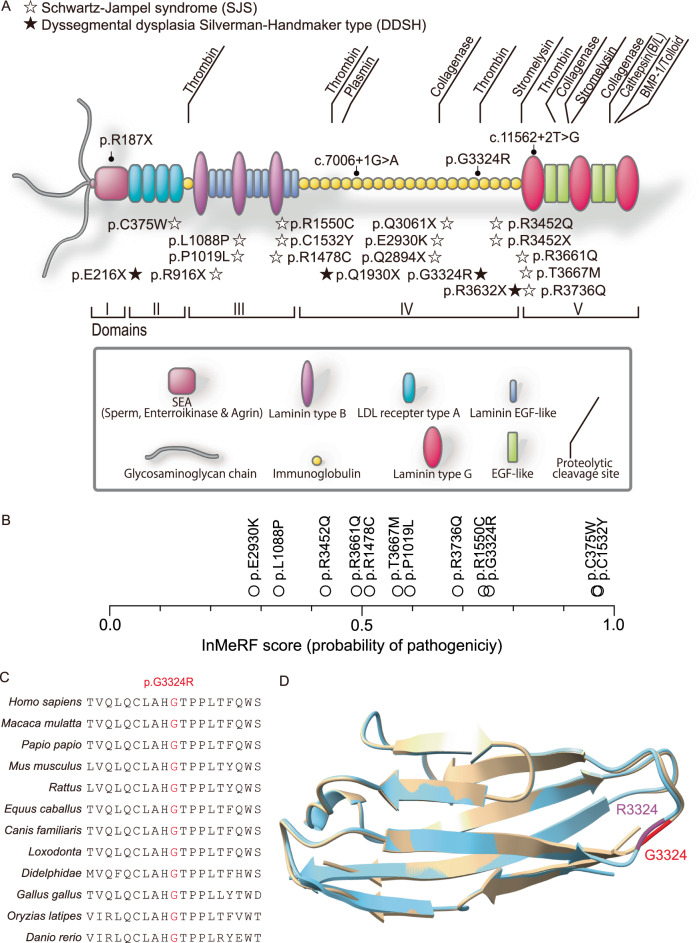
**A** Domain structure of perlecan. Previously reported pathogenic SNVs identified in *HSPG2* in SJS and DDSH (Table 1) are indicated by open and closed stars, respectively. Previously reported splicing variants and indels are not indicated. Pathogenic SNVs identified in this communication (Table 2) are indicated by circles above the domain structure. **B** InMeRF scores of the 11 missense variants in SJS (Table 1) and p.G3324R in the current report. **C** Alignment of G3324 of perlecan orthologs. **D** Overlaid structures of the wild-type and mutant immunoglobulin I-set domains of perlecan at codons 3300 to 3383 (NP_00127878.1) that were predicted by AlphaFold2. The wild-type domain with G3324 (red) is indicated in brown ribbons. The mutant domain with R3324 (magenta) is indicated in light blue ribbons

A total of 62 genes including *HSPG2* were annotated with skeletal dysplasia in HGMD Pro 2020 (Supplementary Table S2), and pathogenic variants that could account for the patients’ phenotypes were observed only in *HSPG2* in all the five patients. Whole-genome and exome sequencing revealed four biallelic pathogenic variants in *HSPG2* in five patients with DDRD (Table 2). Two patients (DDRD_P02 and P03) carried homozygous variants, whereas the other three patients (DDRD_P04, P06, and P07) carried compound heterozygous variants. All the five patients carried c.9970 G > A (NM_005529.7) at position 21,839,005 (GRCh38/hg38) on chromosome 1, which predicted p.G3324R (NP_005520.4) in perlecan domain IV (Fig. 2A). p.G3324R was highly conserved across species (Fig. 2B). p.G3324R was predicted to be pathogenic by InMeRF with a probability score of 0.752, where 1.000 is most pathogenic and 0.000 is least pathogenic (Fig. 2B, Table 2) [13]. p.G3324R has an accession number of rs1294413650 in dbSNP with global minor allelic frequency (GMAF) = 5.7 × 10^−5^ (17/298,038) and Japanese minor allelic frequency (JMAF) = 3.5 × 10^−4^ (10/28,258). p.G3324R was previously reported in a patient in a large cohort comprised of 411 patients with skeletal dysplasia, and was labeled as DDSH (Table 1) [14]. In five available parent samples, the mother of DDRD_P02, the mother and the father of DDRD_P03, and the father but not the mother of DDRD_P06 were heterozygous for p.G3324R (Supplementary Fig. S1).

**Table 2:** Pathogenic variants in *HSPG2* identified in the five patients in the current report

Pt	Coordinate GRCh38	NM_005529.7	NP_005520.4	Hom/C. het^a^	Region/Domain^b^	dbSNP	HGMD	InMeRF score^c,13^	CADD score^c,32^	DANN score^c,33^
2	21,839,005	c.9970 G > A	p.G3324R	Hom	18th immunoglobulin-like region/Domain IV	rs1294413650	CM1821690	0.752	0.927	0.989
3	21,839,005	c.9970 G > A	p.G3324R	Hom	↓	rs1294413650	CM1821690	0.752	0.927	0.989
4	21,839,005	c.9970 G > A	p.G3324R	C. het	↓	rs1294413650	CM1821690	0.752	0.927	0.989
	21,851,790	c.7006 + 1 G > A	splicing	C. het		rs778653296	CS065567	na	na	na
6	21,839,005	c.9970 G > A	p.G3324R	C. het	18th immunoglobulin-like region/Domain IV	rs1294413650	CM1821690	0.752	0.927	0.989
	21,889,996	c.559 C > T	p.R187X	C. het		rs1332584154	-	na	na	na
7	21,839,005	c.9970 G > A	p.G3324R	C. het	18th immunoglobulin-like region/Domain IV	rs1294413650	CM1821690	0.752	0.927	0.989
	21,831,213	c.11562 + 2 T > G	splicing	C. het		-	CS151722	na	na	na

^a^*Hom* homozygous, *C. het* compound heterozygous

^b^Domains are according to RefSeq NM_001291860.2, and are indicated only for missense variants. Please also refer to Fig. 2A for schematic presentation

^c^The most pathogenic score is 1.000 and the least pathogenic score is 0.000 [13, 32, 33]

DDRD_P04 also had a heterozygous c.7006 + 1 G > A (NM_005529.7) at position 21,851,790 (GRCh38/hg38) on chromosome 1 in *HSPG2* intron 54, predicting aberrant splicing by disrupting invariant GT dinucleotides (Table 2, Fig. 2A, and Supplementary Fig. S1). GT dinucleotides at 6 nucleotide downstream to the authentic GT dinucleotides have a low MaxEntScan:5ss score [15] of −6.23, which was much lower than that of 9.40 at the authentic site. Thus c.7006 + 1 G > A is unlikely to activate a cryptic 5′ splice site but is likely to result in frameshifting skipping of the upstream exon (136 bp). c.7006 + 1 G > A has an accession number of rs778653296 in dbSNP with GMAF = 7.4 × 10^−6^ (1/135,190) and JMAF = 0. c.7006 + 1 G > A was previously reported in an SJS patient without functional characterization (Table 1) [7]. The parent’s samples were not available.

DDRD_P06 also had a heterozygous c.559 C > T (NM_005529.7) at position 21,889,996 (GRCh38/hg38) on chromosome 1 in *HSPG2* exon 6, predicting p.R187X (NP_005520.4) in perlecan domain I (Table 2, Fig. 2A, and Supplementary Fig. S1). p.R187X has an accession number of rs1332584154 in dbSNP with GMAF = 6.4 × 10^−6^ (1/156,244) and JMAF = 0. The father was heterozygous for p.G3324R as stated above, and the mother was heterozygous for p.R187X (Supplementary Fig. S1).

DDRD_P07 also had a heterozygous c.11562 + 2 T > G (NM_005529.7) at position 21,831,213 (GRCh38/hg38) on chromosome 1 in *HSPG2* intron 84, predicting aberrant splicing by disrupting invariant GT dinucleotides (Table 2, Fig. 2A, and Supplementary Fig. S1). GT dinucleotides at 20 bp upstream and 33 bp downstream to the authentic GT dinucleotides have low MaxEntScan:5ss scores [15] of −11.07 and −11.54, respectively, which were much lower than that of 6.54 at the authentic 5′ splice site. Thus, c.11562 + 2 T > G is unlikely to activate a cryptic 5′ splice site, but is likely to cause frameshifting skipping of the upstream exon (110 bp). c.11562 + 2 T > G has no accession number in dbSNP, and there is no previous report on this SNV. However, a substitution of T-to-A at the same position (c.11562 + 2 T > A) was previously reported in a patient with cerebral palsy without functional characterization [16]. The parent’s samples were not available.

### Founder haplotype of p.G3324R

**Fig. 3:**
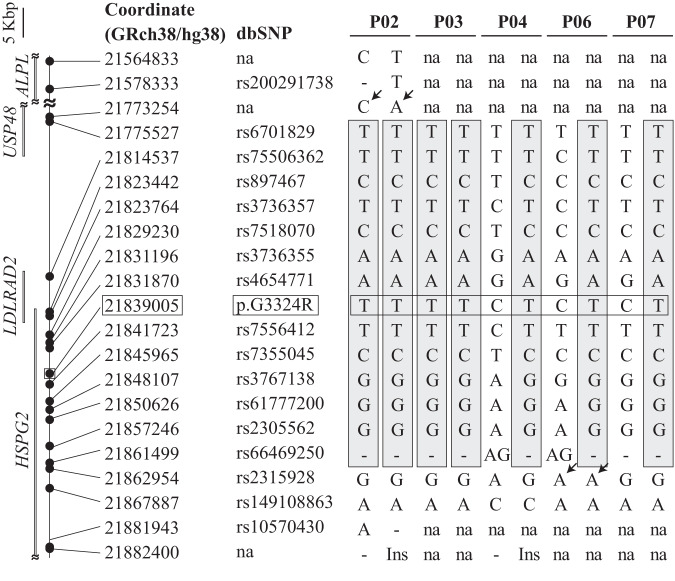
Founder haplotype for p.G3324R of *HSPG2*. The shared haplotype is indicated by shades. Coordinates are drawn to scale. p.G3324R is indicated by rectangles. The upper margin of the shared haplotype is delineated by a heterozygous SNV at position 21,773,254 in DDRD_P02 (arrows). Similarly, the lower margin of the shared haplotype is delineated by the absence of an SNV at 21,862,954 in DDRD_P06 (arrows). na not available, because whole-genome sequencing was performed only in DDRD_P02. Ins, insertion of ACACAC. For heterozygous SNVs, lack of whole-genome- or exome sequencing of the parents disabled us from identifying their allelic distributions and presumptive haplotypes

As all the five patients carried p.G3324R, we looked for a shared haplotype spanning p.G3324R. The shared haplotype was defined as a stretch of SNVs or small indels that were homozygous in DDRD_P02 and P03, and either homozygous or heterozygous in DDRD_P04, P06, and P07. The analysis revealed that an 85,973-bp segment from positions 21,775,527 to 21,861,499 on chromosome 1 (GRCh38/hg38) were shared in the five patients. The margin at the *p*-terminal side was determined by the presence of a heterozygous SNV in DDRD_P02, and that at the *q*-terminal side was determined by the absence of an SNV in DDRD_P06, which was present in the other four patients (arrows in Fig. 3). All SNVs and small indels in 5,000,000 bp up- and downstream of p.G3324R are collated in Supplementary Table S3. Representative SNVs and small indels in the shared haplotype and flanking SNVs are schematically shown in Fig. 3.

## Discussion

Perlecan is a huge extracellular matrix (ECM) proteoglycan (~500 KDa) with five domains and three heparan sulfate chains at the N-terminal (Fig. 2A) [17]. Domain I contains three Ser-Gly-Asp attachment sites for heparan sulfate chains, and one sperm, enterokinase, and agrin homology (SEA) module [18, 19]. Domain II has four cysteine-rich modules [low-density lipoprotein (LDL) receptor type A] [20]. Domain III has three laminin type B modules and eight laminin epidermal growth factor (EGF)-like modules [21]. Domain IV is the largest domain with twenty-one immunoglobulin-like repeats [22]. Domain V is comprised of three laminin type G modules and four EGF-like modules [22]. Perlecan binds to the other ECM and transmembrane proteins like collagen IV, laminin-1, β1 integrin, α dystroglycan, and acetylcholinesterase [5, 23]. Perlecan plays critical roles in the development and remodeling of cartilage, bone, and heart; angiogenesis; and blood brain barrier, as well as wound healing [24–27].

We reported five patients with DDRD carrying p.G3324R in perlecan encoded by *HSPG2* either in a homozygous (DDRD_P02 and P03) or heterozygous (DDRD_P04, P06, and P07) manner. Pathogenic variants on another allele in heterozygous patients were either a nonsense variant (DDRD_P06) or splicing variants (DDRD_P04 and P07), both of which predicted truncated proteins. The five patients had a shared haplotype spanning an 85,973-bp segment (Fig. 3). The *p*-terminal end of the shared haplotype was determined by a heterozygous SNV in DDRD_P02 who was homozygous for p.G3324R. As whole-genome sequencing was performed only in DDRD_P02, this SNV was not sequenced in the other patients. Thus, the other four patients may share an extended haplotype. Similarly, the *q*-terminal end of the shared haplotype was determined by lack of an SNV in DDRD_P06, which was present in the other four patients. Thus, the other four patients may share an extended haplotype. In addition, as the allelic allocations of SNVs and small indels could not be determined in heterozygous DDRD_P04, P06, and P07, the shared haplotype might be shorter than predicted in Fig. 3. DDRD_P02 and P03 were homozygous for p.G3324R, but the skeletal dysplasia other than short long bones became apparent at 31 and 19 weeks of gestation, respectively. Presence of an unidentified modifier gene or an unidentified environmental factor might have accounted for the different disease severities. In any of the five patients, no consanguinity was documented. Similarly, no shared ancestor was noted in any pair of patients. However, the presence of a shared haplotype in five patients is likely to represent identity-by-descent that arose from a single ancestor.

p.G3324R in *HSPG2* was previously reported homozygously in a patient with DDSH [14]. The patient was a 7-year-old girl who was born at full term via normal spontaneous vaginal delivery. The parents were cousins. The patient had cervical kyphosis since birth that progressed over time and resulted in severe cervical canal stenosis and quadriplegic paralysis. The patient also had respiratory complications that led to tracheostomy, and she became respirator dependent. The patient underwent posterior cervical vertebrae repair with no improvement. Milestones other than the motor delay were appropriate for age. Her dysmorphic features included a small mouth, long eyelashes, brachycephaly, micrognathia, cervical kyphosis, and short lower limbs. Her weight and height were below the 3rd percentile. Skeletal examinations showed kyphosis of the cervical spine, platyspondyly, biphasic scoliosis, irregular margins of multiple vertebrae, decreased disc spaces, shorted long bones of the lower limbs, bilateral broadening of the metaphyses and epiphysis of the long bones of the upper and lower limbs, bilateral broadening of the metacarpals and the phalanges, bilateral broadening of the metaphyses of metatarsals and right foot metatarsus adductus. Although the label of DDSH was given to this patient, the nonlethal clinical course and the mild to moderate skeletal dysplasia were consistent with the diagnosis of DDRD.

DDSH and DDRD share similar skeletal phenotypes, while patients with DDSH and DDRD are lethal and nonlethal, respectively. No definite threshold has been proposed to delineate lethal and nonlethal phenotypes. Eight pathogenic variants in *HSPG2* in DDSH have been reported in ten patients in six articles (Table 1) [6, 14, 28–31]. In the ten patients, pregnancy was terminated in five fetuses [6, 28, 29]; a patient was stillborn [29]; and two patients died immediately [6] and two weeks [30] after birth. Details were not documented in the ninth patient [31]. The last patient was the 7-year-old girl stated above [14]. Except for the 7-year-old girl, no patients survived more than two weeks after birth. The prevalence of DDSH and DDRD remains undetermined. As genetic diagnosis of surviving DDRD patients is likely to have a higher clinical significance than that of deceased DDSH patients, DDRD patients are predicted to be subjected to genetic diagnosis. Nevertheless, no pathogenic variants have been reported in DDRD. Thus, the prevalence of DDRD may be less than that of DDSH, which might have prevented us from identifying the genetic cause of DDRD.

Eleven missense variants have been reported in *HSPG2* in SJS (Table 1). We recently developed InMeRF, a tool to predict the pathogenicity of missense variants [13]. InMeRF is comprised of 150 random forest models, where each model is dedicated to predicting the pathogenicity of each amino acid substitution. InMeRF outperformed 25 previously reported prediction tools. We indicated CADD scores [32] and DANN scores [33] in Table 1, but only InMeRF scores will be used in the following discussion. The mean and SD of InMeRF scores of the eleven missense variants in SJS were 0.598 ± 0.227, whereas the InMeRF score of p.G3324R was 0.752 (Fig. 2B, Table 1). Thus, p.G3324R may be more deleterious than most of the pathogenic missense variants in SJS. In addition, two (p.E2930K and p.R3452Q) of the eleven missense variants in SJS, as well as p.G3324R in DDRD, were located in a stretch of 21 immunoglobulin-like regions from codons 1695 to 3655 (domain IV), whereas the other nine missense variants were at different perlecan domains (Fig. 2A ad Table 1). Although the two SJS missense variants still exist in domain IV, a specific site or a specific amino acid substitution in domain IV may be more vulnerable to amino acid substitution than those in the other domains. In contrast to SJS, no missense variant was reported in DDSH except for the 7-year-old girl stated above (Table 1). It was previously proposed that differences in the amount of functional perlecan in the extracellular matrix, as well as the affected domains, account for the differences in SJS and DDSH [7, 34]. An intermediate amount of functional perlecan that is lower than that in SJS but is higher than that in DDSH, as well as domain-specific derangement, may cause DDRD. However, most of the 18 splicing variants in SJS and one of the two splicing variants in DDSH were not functionally characterized, and the ratios of normally spliced transcript remain undetermined. Similarly, the two inframe variants in SJS were not functionally characterized. In addition, the phenotypes are determined by two alleles of *HSPG2*, and the estimation of a clinical phenotype only by a hemiallelic missense variant is not appropriate and is misleading. Nevertheless, the pathogenicity of variants in *HSPG2* may determine which of SJS, DDRD, or DDSH a patient will develop. In addition, we observed mild facial and limb myotonia in DDRD_P04, which are typical features of SJS. Although we did not examine muscle hyperexcitability in the other four patients, DDRD and SJS may share overlapping muscle phenotypes. However, in DDRD, compared to skeletal dysplasia, abnormal muscle hyperexcitability is unlikely to cause disability and is underestimated.

Lack of genetic variants of DDRD in OMIM and ClinVar was unexpected for us. *HSPG2* is a huge gene spanning 115,067 bp on chromosome 1, and is comprised of 97 exons with the maximum mRNA length of 14,341 bp. The size of *HSPG2* and the rarity of DDRD patients are likely to have prevented us from identifying the genetic cause of DDRD. Alternatively, as DDSH and DDRD are expected to be allelic disorders [35], the identification of individual *HSPG2* variant(s) in DDRD may not have been reported due to publication bias. Rapid development of massive parallel sequencing techniques along with lowering the sequencing cost, as well as the development of dependable evaluation tools of SNVs, will enable us to identify more variants in DDRD. In addition, the identification of pathogenic variants in SJS, DDRD, and DDSH will also disclose the molecular organization of perlecan.

### Supplementary information

Supplementary Figure S1

Supplementary Table S1

Supplementary Table S2

Supplementary Table S3

Supplementary Figure Legend
