# Supplementary material for: Dyssegmental dysplasia Rolland–Desbuquois type is caused by pathogenic variants in HSPG2 - a founder haplotype shared in five patients
Source: J Hum Genet. 2024 Feb 29;69(6):235–44. doi: 10.1038/s10038-024-01229-6 (PMC11126378; doi:10.1038/s10038-024-01229-6)
Supplement: Supplementary file 2 — Supplementary Table S1 [file 10038_2024_1229_MOESM2_ESM.docx]

**Supplementary Table S1. Primer sequences for Sanger sequencing**

| Pathogenic variant | Primer sequence (5'->3') |
| --- | --- |
| HSPG2 c.9970G>A |  |
| Forward primer | GCTGCTTACCTTGGACGAGC |
| Reverse primer | ACACTGGATCCAACGACCTG |
| *HSPG2* c.7006+1G>A |  |
| Forward primer | TCATCTCAGGAGCATCTACAACTCTC |
| Reverse primer | TCCTCCAGTCCCATACCTGG |
| *HSPG2* c.559C>T |  |
| Forward primer | CCTATGGGATGAGTCGCTGTG |
| Reverse primer | GTCAGTGCTCAGTAAACAGTTGCTC |
| *HSPG2* c.11562+2T>A |  |
| Forward primer | TGGATCTGAACGAGGAACTCTACC |
| Reverse primer | AGACGCACACGTAGCTGCTG |
