# Supplementary material for: Dyssegmental dysplasia Rolland–Desbuquois type is caused by pathogenic variants in HSPG2 - a founder haplotype shared in five patients
Source: J Hum Genet. 2024 Feb 29;69(6):235–44. doi: 10.1038/s10038-024-01229-6 (PMC11126378; doi:10.1038/s10038-024-01229-6)
Supplement: Supplementary file 3 — Supplementary Table S2 [file 10038_2024_1229_MOESM3_ESM.docx]

**Supplementary Table S2. Sixty-two genes annotated with skeletal dysplasia and their 72 phenotypes in HGMD Pro 2020**

| **Gene** | **Disease** |
| --- | --- |
| *ACVR1* | Skeletal dysplasia |
| *ALG9* | Skeletal dysplasia, polycystic kidneys & multiple malformations |
| *ATR* | Skeletal dysplasia |
| *B3GAT3* | Skeletal dysplasia |
| *B4GALT7* | Skeletal dysplasia, perinatal lethal |
| *C2CD3* | Skeletal dysplasia |
| *CHRNG* | Skeletal dysplasia |
| *CHST3* | Skeletal dysplasia, with craniosynostosis |
| *COL11A1* | Skeletal dysplasia |
| *COL1A1* | Skeletal dysplasia |
| *COL1A2* | Skeletal dysplasia |
| *COL27A1* | Global developmental delay, failure to thrive, dysmorphic facies, skeletal dysplasia and genitourinary abnormalities |
| *COL2A1* | Platyspondylic skeletal dysplasia, Torrance |
| *COL2A1* | Skeletal dysplasia |
| *CSF1R* | Skeletal dysplasia and brain malformation with calcifying leukoencephalopathy |
| *CSGALNACT1* | Skeletal dysplasia and joint laxity |
| *CSGALNACT1* | Skeletal dysplasia with advanced bone age |
| *CTNNB1* | Sclerosing skeletal dysplasia with adrenocortical neoplasia |
| *CYP26B1* | Skeletal dysplasia |
| *DIP2C* | Skeletal dysplasia |
| *EXTL3* | Neuro-immuno-skeletal dysplasia syndrome |
| *EXTL3* | Skeletal dysplasia, immune deficiency & developmental delay |
| *FGFR3* | Skeletal dysplasia |
| *FLNA* | Heterotopia, periventricular with skeletal dysplasia |
| *FLNB* | Skeletal dysplasia |
| *FLNB* | Skeletal dysplasia with 46,XY gonadal dysgenesis |
| *GDF5* | Skeletal dysplasia |
| *HHAT* | Microcephaly, skeletal dysplasia & cerebellar vermis hypoplasia |
| *HSPG2* | Skeletal dysplasia, osteoporosis and flexion contracture |
| *IFT122* | Skeletal dysplasia |
| *INPPL1* | Skeletal dysplasia |
| *JAG1* | Cardiac & skeletal dysplasia |
| *KMT2D* | Skeletal dysplasia |
| *LBR* | Greenberg skeletal dysplasia |
| *LBR* | Skeletal dysplasia |
| *LIFR* | Skeletal dysplasia |
| *MAB21L2* | Bilateral anophthalmia, intellectual disability & rhizomelic skeletal dysplasia |
| *MAGEL2* | Moderate intellectual disability, speech delay, short stature, skeletal dysplasia, failure to thrive. |
| *MBTPS1* | Skeletal dysplasia |
| *MIR140* | Skeletal dysplasia |
| *NANS* | Developmental delay & skeletal dysplasia, infantile-onset |
| *NEK9* | Skeletal dysplasia, lethal |
| *NEPRO* | Macrocephaly, hypoplastic maxilla & skeletal dysplasia |
| *NEPRO* | Skeletal dysplasia with cartilage hair hypoplasia |
| *PAN2* | Skeletal dysplasia |
| *PGM3* | Congenital disorder of glycosylation with severe immunodeficiency & skeletal dysplasia |
| *PGM3* | Severe combined immunodeficiency with bone marrow failure, skeletal dysplasia and congenital malformations |
| *PHEX* | Short stature and bowing of both legs suggestive of skeletal dysplasia |
| *PISD* | Skeletal dysplasia, cataracts and white matter changes |
| *POC1A* | Primordial dwarfism, skeletal dysplasia, facial dysmorphism, extreme insulin resistance and fatty liver. |
| *POP1* | Short stature with mild skeletal dysplasia |
| *POP1* | Skeletal dysplasia |
| *PRG4* | Skeletal dysplasia |
| *PRKAR1A* | Skeletal dysplasia, developmental delay, hypotonia, facial dysmorphism and absent speech |
| *PTH1R* | Eiken skeletal dysplasia |
| *PTH1R* | Eiken skeletal dysplasia with pseudoepiphyses in the hands and primary failure of tooth eruption |
| *RILPL2* | Skeletal dysplasia, speech delay, mototr delay, learning disability |
| *RIN1* | Skeletal dysplasia |
| *SBDS* | Multiorgan involvement (liver, kidney, hemopoethic system), skeletal dysplasia, growth failure, and complex I deficiency |
| *SETBP1* | Skeletal dysplasia, dysmorphic features |
| *SLC10A7* | Skeletal dysplasia with amelogenesis imperfecta |
| *SLC26A2* | Skeletal dysplasia, foetal |
| *SLC35A3* | Skeletal dysplasia |
| *SUCO* | Skeletal dysplasia |
| *TRIP11* | Fetal abnormalities & skeletal dysplasia |
| *TRIP11* | Skeletal dysplasia |
| *TRPV4* | Skeletal dysplasia & peripheral neuropathy |
| *TRPV6* | Bone undermineralisation and skeletal dysplasia |
| *TTC28* | Skeletal dysplasia with multiple joint dislocation |
| *WDR35* | Hepatic cysts with ductal malformation, polycystic kidney with renal failure, skeletal dysplasia & cerebellar hypoplasia |
| *WNT3A* | Skeletal dysplasia |
| *XYLT1* | Skeletal dysplasia, short limb, neonatal |
