# Supplementary material for: Dyssegmental dysplasia Rolland–Desbuquois type is caused by pathogenic variants in HSPG2 - a founder haplotype shared in five patients
Source: J Hum Genet. 2024 Feb 29;69(6):235–44. doi: 10.1038/s10038-024-01229-6 (PMC11126378; doi:10.1038/s10038-024-01229-6)
Supplement: Supplementary file 5 — Supplementary Figure Legend [file 10038_2024_1229_MOESM5_ESM.docx]

**Supplementary Figure S1.** Pedigrees and sequencing chromatograms. **(A)** Five pedigrees with DDRD. DDRD patients with biallelic pathogenic variants in *HSPG2* are indicated by arrows and shades. Asymptomatic parents with hemiallelic pathogenic variants are indicated by half shades. Small open symbols indicate that no DNA was available and the that the individual was asymptomatic. **(B)** Sequencing chromatograms showing pathogenic variants in *HSPG2* in patients and their available parents.
